# Salmeterol plus fluticasone propionate versus fluticasone propionate plus montelukast: a randomised controlled trial investigating the effects on airway inflammation in asthma

**DOI:** 10.1186/1465-9921-8-67

**Published:** 2007-09-27

**Authors:** Ian Pavord, Ashley Woodcock, Debbie Parker, Leanne Rice, the SOLTA study group

**Affiliations:** 1 Glenfield Hospital, Leicester, UK; 2 Wythenshawe Hospital, Manchester, UK; 3 GlaxoSmithKline UK, Uxbridge, UK

## Abstract

**Background:**

Few studies have compared treatment strategies in patients with asthma poorly controlled on low dose inhaled corticosteroids, and little is known about the effects of different treatments on airway inflammation. In this double-blind, placebo-controlled, parallel group study, we compared the effects of salmeterol plus fluticasone propionate (FP) (Seretide™; SFC) and FP plus montelukast (FP/M) on sputum inflammatory markers, airway responsiveness, lung function, and symptoms in adult asthmatics.

**Methods:**

Sixty-six subjects were randomised to SFC or FP/M for 12 weeks. The primary outcome was changes in neutrophil, eosinophil, macrophage, lymphocyte, and epithelial cell levels in induced sputum. Additional outcomes included the change in other sputum markers of airway inflammation, airway responsiveness, symptom control, and lung function.

**Results:**

Both treatments had no significant effect on induced sputum inflammatory cells, although there was a trend for a reduction in sputum eosinophils. Both treatments significantly improved airway responsiveness, whereas SFC generally led to greater improvements in symptom control and lung function than FP/M. FP/M led to significantly greater reductions in sputum cysteinyl leukotrienes than SFC (treatment ratio 1.80; 95% CI 1.09, 2.94).

**Conclusion:**

Both treatments led to similar control of eosinophilic airway inflammation, although PEF and symptom control were better with SFC.

**Study number:**

SAM40030 (SOLTA)

## Background

Asthma is a complex disorder characterised by airway hyperresponsiveness and variable airflow obstruction in association with eosinophilic airway inflammation [1]. Treatment with an inhaled corticosteroid (ICS) is usually the first line of therapy [1]. For patients whose asthma is poorly controlled on ICS therapy alone, current management guidelines recommend add-on therapy with a long-acting β_2_-agonist, or alternatively a leukotriene receptor antagonist [2,3]. A recent Cochrane systematic review of eight randomised controlled trials has shown that the addition of a long-acting β_2_-agonist (salmeterol or formoterol) to existing ICS therapy leads to more effective asthma control than addition of a leukotriene receptor antagonist (montelukast or zafirlukast) [4]. The combination of a long-acting β_2 _agonist and an ICS led to significant reductions in the risk of exacerbations, night awakenings, and rescue medication use, and significant improvements in lung function, symptoms, and quality of life [4].

The exact relationship between the underlying inflammatory processes and the clinical expression of asthma is complex and not fully understood. Cross-sectional studies have shown only a weak correlation at best between the severity of airway inflammation, measured by inflammatory cell counts or nitric oxide exhalation, and airway hyper-responsiveness, severity of symptoms, or abnormalities in lung function [5-7]. There is limited information on the effects of long-acting β_2_-agonists and leukotriene receptor antagonists on eosinophilic airway inflammation in asthma. A better understanding of these effects might inform our understanding of the relationship between inflammation and the clinical expression of asthma. The objective of this study was to compare the effects of the combination of salmeterol and the ICS fluticasone propionate (FP) (Seretide™; SFC) versus adding montelukast to FP therapy (FP/M) on airway inflammatory markers, and to relate these effects to changes in airway responsiveness, lung function, and symptom control.

## Methods

### Study population

Asthma patients aged 18–50 years, who were non-smokers and receiving a stable dose of up to 400 μg of beclometasone dipropionate (BDP) a day or equivalent ICS, but requiring further therapy, were recruited. Additional inclusion criteria included likelihood of compliance with the protocol requirements and ability, following instruction, to use an Accuhaler and mini-Wright peak flow meter. The criteria for randomisation were: a baseline forced expiratory volume in 1 second (FEV_1_) of 61–85% of the predicted normal value; and a PC_20 _< 8 mg/ml with methacholine challenge. In addition, all patients had to have at least one of the following: diary card recording of symptoms (score of one or more for day and night combined) on ≥ 4 of the last seven days of the run-in period; recorded use of relief medication (inhaled Ventolin) on ≥ 2 different days during the last seven days of the run-in period; and a period variation in peak expiratory flow (PEF) of ≥ 10% over the last seven days of the run in period. Patients who did not meet the latter three criteria were able to repeat the run-in period once more. Patients who fulfilled any of the following exclusion criteria did not take part in the study: were taking or had previously taken additional asthma medication, other than an ICS or short acting β_2_-agonist or oral corticosteroids in the last three months; acute respiratory infection or exacerbation of asthma within four weeks of screening, any additional underlying lung disease, or any significant disease warranting exclusion; hospitalisation or emergency treatment (for > 24 hours) for acute asthma in the last 12 months; were a smoker, had smoked in the last six months, or had a smoking history of 10 pack years or more; pregnant or lactating women, or women of child-bearing potential not using adequate contraception; evidence of alcohol, drug, or solvent abuse; hypersensitivity to any component of the study formulations, or taking medication contraindicated in combination with the study formulations; and previous entry to the study or receipt of any investigational drugs within four weeks of screening.

The study was approved by the ethics committee appropriate to each centre before any subjects were enrolled at that centre, and was conducted in accordance with the Declaration of Helsinki. All subjects gave written informed consent before entering the study. The study ran from May 2001 to August 2002.

### Study design and treatments

This was a multicentre, randomised, double-blind, single-dummy, parallel group study comparing SFC with FP/M. After an initial screening visit, subjects participated in a 2-week run-in period to determine eligibility for randomisation based on a number of pre-specified criteria. During the run-in period, subjects continued to take their normal dose of ICS and recorded details of symptoms and relief medication use on diary cards. During the treatment period, subjects discontinued their normal asthma medication and were consecutively randomised according to a pre-defined randomisation list to either Seretide 50 (GlaxoSmithKline; salmeterol 25 μg/FP 50 μg) metered dose inhaler (MDI) two puffs twice daily (bd) (SFC) plus a placebo to montelukast once daily (od) at night or FP 50 μg MDI (Flixotide™, GlaxoSmithKline) two puffs bd plus montelukast 10 mg od at night (FP/M) for 12 weeks, with an interim clinic visit at six weeks. Treatment allocation was concealed from the subject, pharmacist, and investigator.

### Outcome measures

The primary outcome measure was the levels of inflammatory cells (neutrophils, eosinophils, macrophages, and lymphocytes) and epithelial cells in induced sputum. Secondary outcome measures were the levels of the inflammatory markers cysteinyl leukotrienes (C-LT), histamine, and interleukin-8 (IL-8) in induced sputum, bronchial responsiveness to methacholine, the percentages of symptom-free days and nights, the percentages of rescue medication-free days and nights, and lung function measurements (FEV_1 _measured by spirometer and patient assessed morning and evening PEF). The safety outcome measure was adverse events (AEs).

### Induced sputum collection and analysis

Subjects inhaled increasing concentrations (3%, 4%, 5%) of 5 ml nebulised pyrogen-free hypertonic saline, and after each inhalation were encouraged to cough sputum into a plastic container [8]. If FEV_1 _fell by > 10% or 200 ml but < 20% or 400 ml during the initial inhalation, then 3% saline was used for the second and third inhalations. If FEV_1 _fell by > 20% or 400 ml or significant symptoms developed, nebulisation was stopped and the subject was treated with a short-acting β_2_-agonist. The sputum samples were analysed for the following inflammatory markers: neutrophils; eosinophils; lymphocytes; epithelial cells; histamine, as a marker of mast cell activation; C-LT, as a marker of mast cell and eosinophil activation; and IL-8, as a marker of neutrophil activation. Sputum IL-8, C-LT and histamine were measured using standard ELISA kits (BD Pharmagen, Immunotech and Cayman chemicals respectively). These assays have been previously validated for use in sputum supernatants [8]. The sensitivity levels of the assay were 13 × 10^-3^, 0.8 × 10^-3^, 50 × 10^-3 ^ng/ml for C-LTs, IL-8 and histamine. The intra-assay coefficient of variability was 5–10% and the interassay coefficient of variability 3–15% across a range of concentrations of mediators measured.

### Methacholine challenge

After measurement of baseline FEV_1_, subjects were administered doubling doses of methacholine chloride (0.025–25 mg/ml; manufacturer: methapharm inc) every five minutes followed by measurement of FEV_1_. The procedure was continued until FEV_1 _fell by ≥ 20% or until the highest concentration of methacholine chloride was reached. The cumulative provocation concentration of methacholine causing a 20% reduction in FEV_1 _(PC_20_) was determined by linear interpolation between log concentrations enclosing a 20% reduction in FEV_1_. The change in methacholine PC_20 _was expressed in doubling concentrations calculated for each subject using the following formula:

(log_10 _PC_20 _end of treatment - log_10 _PC_20 _baseline)/log_10_(2)

### Statistical methods

As this was an exploratory study, no formal sample size calculation was carried out: sample size was, therefore, limited by subject availability.

All summaries and analyses are for the intention-to-treat population (all subjects receiving at least one dose of the study drug). No imputations were performed for missing data. Therefore if data were missing for either baseline or one of the timepoints, it was not possible to calculate a change from baseline. However, all available data have been used for relevant summaries. The change from baseline in levels of the primary inflammatory markers and IL-8 was analysed within each treatment group using the Wilcoxon signed rank test, owing to the non-normality of the data. Between group comparisons for the primary inflammatory markers, IL-8, symptom-free days and nights, and rescue-free days and nights were analysed using the van Elteren extension to the Wilcoxon rank sum test, adjusting for baseline values. The confidence interval (CI) was calculated using the Hodges-Lehmann method, ignoring the adjustment for baseline and pooling all treatment differences.

The results of analyses with log transformed values (C-LT and histamine) and log_10 _transformed PC_20 _values are back transformed and presented as ratios of geometric means or doubling dose changes rather than log differences. The ratio of SFC to FP/M was also calculated and is referred to as the treatment ratio. Within and between group differences were analysed using the paired t-test and analysis of covariance (ANCOVA) adjusting for baseline values, respectively. Between treatment comparisons for change in FEV_1 _and morning and evening PEF were also analysed using ANCOVA, adjusting for baseline values, age, sex, and height. Age, sex and height were adjusted for in these analyses to balance out differences expected. All statistical tests were two-sided and significance was accepted at the 5% level.

## Results

### Demographic and baseline characteristics

**Figure 1:**
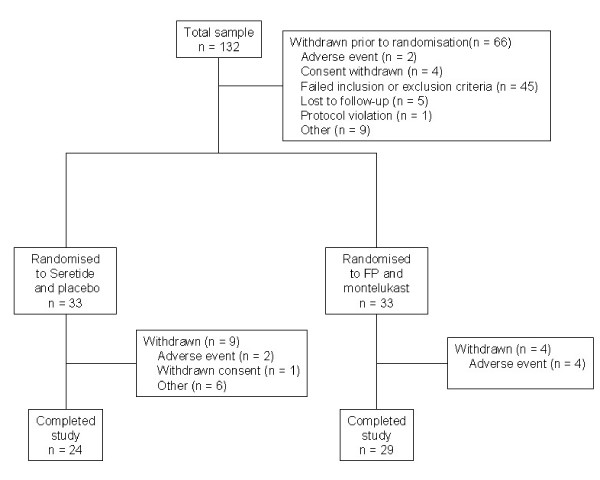
**Flow of subjects through the study**. FP, fluticasone propionate; n, number

Of the 132 subjects enrolled into the study, 66 were randomised to treatment, 33 in each treatment group. The flow of subjects through the study is shown in Figure 1. "Other" reasons for withdrawal from the treatment period in the SFC arm were: did not attend for a visit (2 subjects); failed Methacholine challenge (2 subjects); sputum sample inadequate (1 subject); study medication discarded in error (1 subject). The treatment groups were well matched for baseline characteristics (Table 1). Sixty-five subjects (98%) were taking an ICS on entry to the study, which was BDP in the majority of cases (62/65; 95%).

**Table 1:** Demographic and baseline characteristics

	**SFC (n = 33)**	**FP/M (n = 33)**
Mean age in years (SD)	36.3 (8.11)	34.4 (7.71)
No of females (%)	18 (55%)	14 (42%)
Mean duration of asthma in years (SD)	21.1 (12.7)	18.4 (9.7)
Mean AM PEF (L/min) (SD)	412 (95)	422 (103)
Mean % predicted mean AM PEF (SD)	84 (12)	83 (14)
Mean clinic FEV_1 _(L) (SD)	2.65 (0.77)	2.76 (0.60)
% predicted clinic FEV_1 _(SD)	75 (9)	76 (7)
Geometric mean PC_20 _(mg/ml)	0.28	0.32
Median dose of pre-study ICS (BDP equivalent) (mcg) (range)	400 (200 to 400)	400 (100 to 400)

AM, morning; FEV_1_, forced expiratory volume in 1 second; FP/M, fluticasone propionate plus montelukast; n, number; PEF, peak expiratory flow; SD, standard deviation; SFC, salmeterol plus fluticasone propionate

### Induced sputum inflammatory markers

**Figure 2:**
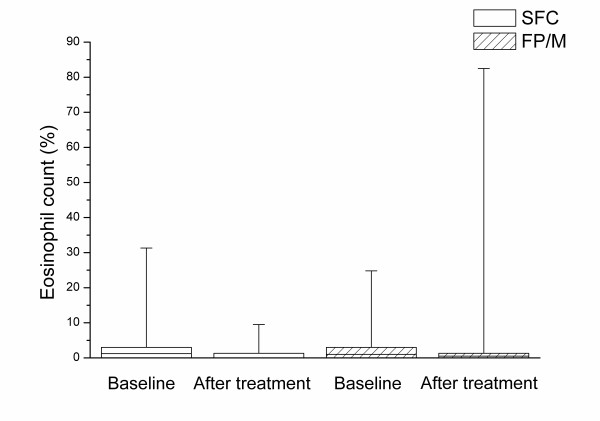
**Eosinophil count in induced sputum before and after treatment**. The box is determined by the first and third quartile and the whiskers are determined by the maximum and minimum. The horizontal line represents the median value. SFC, salmeterol plus fluticasone propionate; FP/M, fluticasone propionate plus montelukast

**Figure 3:**
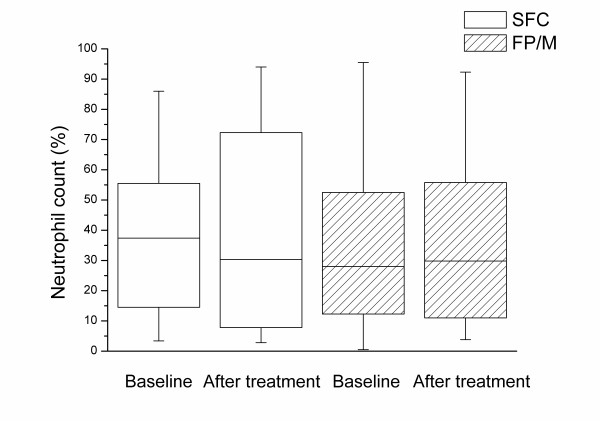
**Neutrophil count in induced sputum before and after treatment**. The box is determined by the first and third quartile and the whiskers are determined by the maximum and minimum. The horizontal line represents the median value. SFC, salmeterol plus fluticasone propionate; FP/M, fluticasone propionate plus montelukast.

Satisfactory sputum samples were obtained at baseline from 79% (26/33) of subjects randomised to SFC and 82% (27/33) of subjects randomised to FP/M. At end of treatment these percentages were 52% SFC (17/33) and 58% FP/M (19/33). The percentage of eosinophils decreased relative to baseline in each treatment group with no significant difference between treatment groups (Table 2 and Figure 2). There was a slight trend for the sputum neutrophil count to decrease with SFC although the change was not significantly different from that seen with FP/M (mean difference in change in neutrophil differential count [SFC – FP/M] -9.4%; 95% CI -26.5%, 6.40%; p = 0.13; Table 2 and Figure 3). There were no other statistically significant changes in sputum cell counts (Table 2).

**Table 2:** Change from baseline in levels of airway inflammatory markers

**Marker**	**SFC (n = 33)**		**FP/M (n = 33)**		**Treatment difference (95% CI)**
	**Baseline**	Change* (95% CI)	**Baseline**	Change* (95% CI)	

Primary markers (median)					
Neutrophils (%)	37.40	-2.30 (-16.80, 14.70)	28.00	3.00 (-7.20, 15.50)	-9.40 (-26.50, 6.40)
Eosinophils (%)	1.20	-0.50 (-2.80, 0.80)	1.00	-0.50 (-1.00, 0.00)	0.20 (-1.50, 1.50)
Lymphocytes (%)	0.00	0.00 (-0.30, 0.50)	0.00	0.00 (0.00, 0.00)	0.00 (-0.30, 0.50)
Macrophages (%)	45.40	4.00 (-10.70, 15.20)	64.00	-0.50 (-8.70, 10.80)	1.70 (-12.60, 17.20)
Epithelial cells (%)	3.40	0.00 (-4.30, 8.00)	6.00	0.00 (-3.50, 2.50)	2.20 (-2.80, 9.00)
Secondary marker (median)					
IL-8 (ng/ml)	3.80	0.00 (-0.29, 5.55)	2.99	0.00 (-5.32, 1.11)	3.85 (-0.32, 8.08)

* median change from baseline

CI, confidence interval; FP/M, fluticasone propionate plus montelukast; n, number; SFC, salmeterol plus fluticasone propionate

The mean levels of C-LT and histamine decreased in both treatment groups relative to baseline, although a significant treatment effect was only seen for C-LT in the FP/M group (treatment ratio for C-LT 1.80; 95% CI 1.09, 2.94; p = 0.021; Table 3). Neither treatment had a significant effect on the median IL-8 concentration (Table 3).

**Table 3:** Ratio of mean endpoint to baseline levels for cysteinyl leukotrienes and histamine

**Marker**	**SFC (n = 33)**		**FP/M (n = 33)**		**Treatment ratio (95% CI)**
	**Baseline**	Ratio* (95% CI)	**Baseline**	Ratio* (95% CI)	
		
C-LT (ng/ml)	1.14	0.97 (0.68, 1.37)	0.91	0.54^† ^(0.38, 0.77)	1.80 (1.09, 2.94)^‡^
Histamine (ng/ml)	5.10	0.65 (0.36, 1.18)	5.16	0.95 (0.52, 1.73)	0.69 (0.30, 1.62)

* ratio of endpoint value to baseline value, adjusted for baseline values; † within-group ratio p = 0.005; ‡ between group p = 0.021

CI, confidence interval; FP/M, fluticasone propionate plus montelukast; n, number; SFC, salmeterol plus fluticasone propionate

### Airway responsiveness

Geometric mean PC_20 _significantly increased from 0.28 to 1.02 mg/ml (mean difference 1.74 doubling doses; 95% CI 0.96 doubling doses to 2.52 doubling doses) with SFC and from 0.32 to 1.10 mg/ml with FP/M (mean difference 1.98 doubling doses; 95% CI 0.93 doubling doses to 3.03 doubling doses); there was little difference between treatments (treatment ratio 0.91; 95% CI 0.46, 1.78; p = 0.77).

### Lung function

There was a non-significant trend towards a greater increase from baseline in FEV_1 _with SFC compared with FP/M. Increases in both mean morning and evening PEF were significantly greater after SFC treatment compared with FP/M (Table 4).

**Table 4:** Mean change from baseline in FEV_1_, and morning and evening PEF after treatment

**Variable**	**SFC (n = 33)**		**FP/M (n = 33)**		**True treatment difference (95% CI)**
	**Baseline (mean)**	Change* (mean (95% CI))	**Baseline (mean)**	Change* (mean (95% CI))	

FEV_1 _(L)	2.65	0.41 (0.27, 0.56)	2.76	0.30 (0.16, 0.44)	0.11 (-0.10, 0.32)
Mean Morning PEF (L/min)	411.8	61.9 (48.3, 75.6)	421.5	28.7 (14.7, 42.6)	33.3 (13.6, 52.9)^†^
Mean Evening PEF (L/min)	438.8	41.7 (28.3, 55.1)	439.3	19.0 (5.3, 32.7)	22.7 (3.4, 42.1)^‡^

* adjusted mean change from baseline, adjusted for baseline values, age, sex, and height; † between group p = 0.001; ‡ between group p = 0.022

CI, confidence interval; FEV_1_, forced expiratory volume in 1 second; FP/M, fluticasone propionate plus montelukast; n, number; PEF, peak expiratory flow; SFC, salmeterol plus fluticasone propionate

### Symptoms

There was a trend for greater improvements in symptom scores with SFC compared with FP/M. The median percentage of symptom-free days increased from 14% to 71% and from 29% to 67% after SFC and FP/M treatment, respectively (mean difference in change 13.2%, 95% CI -1.9%, 32.9%, p = 0.064). Similarly, for symptom-free nights, there was an increase from 52% to 89% in the SFC-treated group and from 57% to 82% in the FP/M-treated group (mean difference in change 13.3%; 95% CI -1.5%, 34.5%; p = 0.055). SFC also led to greater increases from baseline in median rescue medication-free days (14% to 73% versus 29% to 70%) and nights (50% to 93% versus 71% to 82%) compared with FP/M, although, after adjusting for baseline values, the treatment effect was only significant for rescue-free nights (treatment difference 16.5%; 95% CI 1.4%, 36.1%; p = 0.01).

### Safety: adverse events

There was no difference in the incidence of AEs between the SFC-and FP/M-treated groups (31 AEs in 19 subjects versus 31 AEs in 21 subjects) and AEs most commonly affected the ear, nose, and throat, lower respiratory and gastrointestinal body systems. Three AEs in three subjects in the SFC-treated group and eight AEs in five subjects in the FP/M-treated group were considered to be causally related to treatment. Two subjects, both treated with SFC, reported serious adverse events (pituitary tumour; haematuria), neither of which were assessed as being related to the study drugs. There were no deaths during the study.

## Discussion

This study compared the effects of treatment with a combination of an ICS (FP) with either a long-acting β_2_-agonist (salmeterol) or a leukotriene receptor antagonist (montelukast) on markers of airway inflammation, lung function, and symptom control in patients with poorly controlled asthma. The results are based on a small exploratory study and should be considered as hypothesis-generating rather than definitive.

The primary outcome was the extent of airway inflammation as determined by the levels of inflammatory cells in sputum. We chose to analyse induced sputum samples as opposed to bronchoalveolar lavage (BAL) or bronchial biopsy as the procedure is less invasive and has been shown to be more sensitive for detecting changes in markers of airway inflammation [9]. We found that sputum eosinophil counts were generally low in those patients with symptomatic asthma already established on treatment with a low or moderate dose ICS. Neither treatment combination led to significant changes from baseline in the primary inflammatory markers. Recent studies suggest reduction in peripheral blood eosinophil counts with the addition of montelukast to ICS therapy in patients with poorly controlled asthma [10-13]. The lack of effect on sputum eosinophils might reflect the low baseline levels.

We did see a significantly greater reduction in the concentration of C-LT with FP/M compared with SFC. Montelukast has no known effect on leukotriene biosynthesis so an effect on C-LT concentrations would not be anticipated. Perhaps montelukast reduces the activation status of inflammatory cells producing C-LT. Neither treatment combination had any effect on the concentration of other inflammatory mediators.

A notable finding of the current study is that most patients with symptomatic asthma despite treatment with low dose ICS had no sputum evidence of eosinophilic airway inflammation. Moreover, the improvement in symptoms, airway responsiveness, and lung function seen with additional salmeterol, and to a lesser extent montelukast, was not associated with any change in the sputum eosinophil count. These observations provide further support for the view that the mechanisms that cause symptoms and abnormal airway function can be disassociated from eosinophilic airway inflammation [5-7]. The residual symptoms and abnormal airway function seen in our patients in the absence of eosinophilic airway inflammation could reflect the effect of permanent structural changes due to airway remodelling, although the beneficial effects of long-acting β_2_-agonists seen in this and other studies [4] suggest that they are, at least partly, due to the presence of a corticosteroid resistant, β_2_-agonist responsive abnormality of airway function.

Generally, SFC treatment led to better symptom control and improved lung function than the FP/M combination, with significantly more rescue medication-free nights and greater increases relative to baseline in morning and evening PEF. These results are consistent with the results of the Cochrane review, which suggested superiority for the long-acting β2-agonist and ICS combination over the combination of a leukotriene receptor antagonist and an ICS, with significantly greater improvements in lung function, symptom control, and quality of life [4]. The findings of the current study are important in that they suggest that the beneficial effects of long-acting β2-agonist and ICS combination over the combination of a leukotriene receptor antagonist and an ICS are also seen in a population who do not have to meet inclusion criteria which include a large acute bronchodilator response. There has been concern that the greater suppression of clinical expression of asthma seen with long acting β2-agonists might be associated with masking of eosinophilic airway inflammation and perhaps a higher risk of serious exacerbations and/or airway remodelling [14]. Our findings clearly indicate that, when given in the form of a combination inhaler, there is no evidence of worsening airway inflammation when salmeterol is added to low dose ICS.

## Conclusion

When added to low dose inhaled FP, neither salmeterol nor montelukast were associated with significant changes in sputum inflammatory cells. The addition of salmeterol to FP produced significantly better symptom control and PEF than the addition of montelukast.

## Competing interests

IP has received payments for lectures and for attending scientific conferences from GSK and Astra Zeneca. He has received an unrestricted research grant of €250,000 from GSK. AW has been reimbursed for chairing and speaking at scientific meetings and has served on advisory boards for GSK, Chiesi, Schering Plough and Novartis. He has received research grants and is a principal investigator on GSK studies. DP has no competing interests. LR is an employee of GSK.

## Authors' contributions

IP was involved in the design of the study and the interpretation of the results, as well as reviewing the manuscript. AW was involved in the conduct of the study, the interpretation of the results and the reviewing of the manuscript. DP was involved in eth analysis of the laboratory samples and the reviewing of the manuscript. LR was involved in the design and statistical analysis of the study as well as coordinating and reviewing the manuscript. All authors read and approved the final manuscript.

## References

[B1] Barnes PJ (2002). The role of inflammation and anti-inflammatory medication in asthma. Respir Med.

[B2] (2002). Global Initiative for Asthma. Global strategy for asthma management and prevention. NHLBI/WHO Workshop Report. NIH Publication number 02-3659. http://www.ginasthma.org.

[B3] (2004). British Thoracic Society and Scottish Intercollegiate Guidelines network. British guidelines on the management of asthma. http://www.sign.ac.uk.

[B4] Ram F, Cates C, Ducharme F (2005). Long-acting β_2 _-agonists versus anti-leukotrienes as add-on therapy to inhaled corticosteroids for chronic asthma. Cochrane Database Syst Rev.

[B5] Chapman ID, Foster A, Morley J (1993). The relationship between inflammation and hyperreactivity of the airways in asthma. Clin Exp Allergy.

[B6] Green RH, Brightling CE, Woltmann G, Parker D, Wardlaw AJ, Pavord ID (2002). Analysis of induced sputum in adults with asthma: identification of subgroup with isolated sputum neutrophilia and poor response to inhaled corticosteroids. Thorax.

[B7] Wardlaw AJ, Brightling CE, Green R, Woltmann G, Bradding P, Pavord ID (2002). New insights into the relationship between airway inflammation and asthma. Clin Sci (Lond).

[B8] Birring SS, Parker D, Brightling CE, Bradding P, Wardlaw AJ, Pavord ID (2004). Induced sputum inflammatory mediator concentrations in chronic cough. Am J Respir Crit Care Med.

[B9] Nocker RE, Out TA, Weller FR, de Riemer MJ, Jansen HM, van der Zee JS (2000). Induced sputum and bronchoalveolar lavage as tools for evaluating the effects of inhaled corticosteroids in patients with asthma. J Lab Clin Med.

[B10] Bjermer L, Bisgaard H, Bousquet J, Fabbri LM, Greening AP, Haahtela T, Holgate ST, Picado C, Menten J, Dass SB, Leff JA, Polos PG (2003). Montelukast and fluticasone compared with salmeterol and fluticasone in protecting against asthma exacerbation in adults: one year, double blind, randomised, comparative trial. BMJ.

[B11] Wilson AM, Dempsey OJ, Sims EJ, Lipworth BJ (2001). Evaluation of salmeterol or montelukast as second-line therapy for asthma not controlled with inhaled corticosteroids. Chest.

[B12] Currie GP, Lee DK, Haggart K, Bates CE, Lipworth BJ (2003). Effects of montelukast on surrogate inflammatory markers in corticosteroid-treated patients with asthma. Am J Respir Crit Care Med.

[B13] Ilowite J, Webb R, Friedman B, Kerwin E, Bird SR, Hustad CM, Edelman JM (2004). Addition of montelukast or salmeterol to fluticasone for protection against asthma attacks : a randomized, double-blind, multicenter study. Ann Allergy Asthma Immunol.

[B14] Mcivor RA, Pizzichini E, Turner MO, Hussack P, Hargreave FE, Sears MR (1998). Potential masking effects of salmeterol on airway inflammation in asthma. Am J Respir Crit Care Med.

